# Efficacy and Toxicity of Weekly Carboplatin and Paclitaxel as Induction or Palliative Treatment in Advanced Esophageal Cancer Patients

**DOI:** 10.3390/cancers11060826

**Published:** 2019-06-13

**Authors:** Femke M. de Man, Ruben A.G. van Eerden, Esther Oomen-de Hoop, Joris N. Veraart, Nadia van Doorn, Leni van Doorn, Ate van der Gaast, Ron H.J. Mathijssen

**Affiliations:** Department of Medical Oncology, Erasmus MC Cancer Institute, 3015 GD Rotterdam, The Netherlands; r.vaneerden@erasmusmc.nl (R.A.G.v.E.); e.oomen-dehoop@erasmusmc.nl (E.O.-d.H.); j.veraart@erasmusmc.nl (J.N.V.); n.vandoorn@erasmusmc.nl (N.v.D.); l.vandoorn@erasmusmc.nl (L.v.D.); a.vandergaast@erasmusmc.nl (A.v.d.G.); a.mathijssen@erasmusmc.nl (R.H.J.M.)

**Keywords:** esophageal cancer, induction treatment, palliative treatment, efficacy, toxicity

## Abstract

Many patients have advanced esophageal cancer at diagnosis. However, the most optimal treatment has not been identified. Therefore, we evaluated a weekly regimen of carboplatin (area under the curve (AUC)) of 4 and paclitaxel at 100 mg/m^2^ as an induction or palliative treatment. All patients with advanced (gastro)esophageal cancer treated with this regimen between 2002–2018 were included. Exclusion criteria were previous radiotherapy or treatment elsewhere. Data on toxicity, response, and survival were collected. Analyses were performed in two groups: induction (iCT) or palliative chemotherapy (pCT). Median progression free survival (PFS) and overall survival (OS) were estimated with the Kaplan–Meier method. A total of 291 patients was included (iCT: 122; pCT: 169). Most patients had T3 carcinoma (iCT: 54%; pCT: 66%) and stage IV disease (iCT: 42%; pCT: 91%). A toxicity grade ≥3 occurred mainly as hematological toxicity (iCT: 71%; pCT: 73%) and gastrointestinal toxicity (iCT: 3%; pCT: 5%). Response rates were 48% (iCT) and 44% (pCT). Esophagectomy or definitive chemoradiotherapy followed in 42% of iCT, resulting in a PFS of 22.1 months (interquartile range (IQR): 12.4–114.2) and OS of 26.8 months (IQR: 15.4–91.7). For pCT, PFS was 8.2 months (IQR: 5.1–14.5) and OS 10.9 months (IQR: 6.5–18.3). This retrospective cohort study demonstrated that weekly carboplatin (AUC4) and paclitaxel (100 mg/m^2^) is a well-tolerated and effective induction or palliative treatment regimen for patients with locally advanced or metastatic disease. Future research should directly compare this treatment regimen with other first-line treatment options to determine its true value for clinical practice.

## 1. Introduction

Esophageal cancer is currently the 8th most common cancer type worldwide; the incidence is still rising, and its mortality is high [1,2,3]. Squamous cell carcinoma (SCC) is the most common histology for esophageal cancer worldwide, though adenocarcinomas (AC) are more dominant in the Western world due to typical welfare risk factors including obesity, smoking, and chronic gastroesophageal reflex resulting in Barrett’s esophagus [1,3,4]. Esophageal cancer occurs three to four times more often in male than in female patients in both histological subtypes [1,3,4]. However, the reason for the lower incidence in women is not completely understood; it has been suggested that female hormones or the different body fat distribution may protect women against this type of tumor [5,6,7]. Furthermore, Bohanes et al. demonstrated that the male sex is an independent adverse prognostic factor for esophageal cancer-specific survival with a shorter survival for men compared to women in both locally advanced and metastatic disease [8]. Other adverse prognostic factors are a performance status of 2 or higher, significant weight loss before diagnosis (i.e., ≥10%), adenocarcinoma as histological subtype, liver or peritoneal metastases, an extensively disseminated disease, and an elevated alkaline phosphatase or lactate dehydrogenase [9,10,11,12]. Anatomic origin (i.e., esophageal, esophageal-gastric junction, or gastric) was not identified as a significant prognostic factor in two studies [9,13], while another study demonstrated that tumors arising in the lower one-third of the esophagus did have a worse survival compared to tumors located in the cervical and upper esophagus [12]. 

Although the prognosis of esophageal cancer has improved over the last decades, the outcome still remains poor with an overall 5-year survival of 20% [2,4]. The improvement in prognosis might be caused by recent advances in the treatment of patients with resectable (gastro)esophageal cancer by the introduction of neoadjuvant chemoradiotherapy such as the CROSS-regimen [14,15]. However, almost half of all patients already have unresectable (gastro)esophageal cancer at diagnosis (i.e., locally advanced tumors or distant metastasis) [16]. For patients with a locally advanced disease, a systemic treatment can be considered in an attempt to downstage the tumor (i.e., induction treatment), which can be followed by surgery or chemoradiotherapy in case of good response. For induction chemotherapy, several treatment regimens are described; most of them are platinum- or fluoropyrimidine-based [17,18,19].

For patients with distant metastases, palliative chemotherapy can be considered [20,21]. Palliative systemic treatment improves survival compared to the best supportive care, yet survival benefit is limited, and toxicity should be taken into account [4,20,21,22]. Many different palliative treatment regimens are described, which are often fluoropyrimidine- or platinum-based doublet or triplet combination regimens [4,20,21]. Triplet regimens might be more effective than doublet regimens; however, the incidence of severe toxicity increases significantly in triplet regimens compared to doublets [21,23]. International guidelines often recommend the combination of a fluoropyrimidine and platinum compound as a first-line treatment in metastatic (gastro)esophageal cancer, but it should be noted that these guidelines are sometimes consensus based. The Dutch Esophagus Cancer Guideline dictates that chemotherapy can be considered in metastatic esophageal cancer but does not recommend one specific treatment regimen [24]. A study of the Netherlands Cancer Registry demonstrated that, in the Netherlands only, up to 69 different palliative treatment regimens are administered in metastatic (gastro)esophageal cancer patients [25]. This clearly demonstrates that the most optimal palliative treatment in esophageal cancer is not well-defined.

Fifteen years ago, our research group performed a phase-1 study of weekly paclitaxel and carboplatin as palliative treatment for patients with metastatic esophageal cancer [26]. This regimen uses the same chemotherapeutic backbone as the CROSS-regimen but is not combined with radiotherapy. As a result, higher dosages are possible with a recommended dose for paclitaxel of 100 mg/m^2^ and carboplatin targeted at an area under the curve (AUC) of 4 mg × min/mL [26]. This weekly regimen appeared to be very tolerable and effective with an overall response rate of 54%. Therefore, this regimen was utilized for most patients with advanced or metastatic (gastro)esophageal cancer at the Erasmus University Medical Center, Rotterdam, The Netherlands. The current analysis describes the efficacy and toxicity of this weekly carboplatin and paclitaxel regimen as an induction or palliative treatment option in a real-world treatment setting. Furthermore, predictive factors for treatment outcome and prognostic factors for survival will be analyzed. 

## 2. Results

### 2.1. Patient, Tumor, and Treatment Characteristics

**Figure 1:**
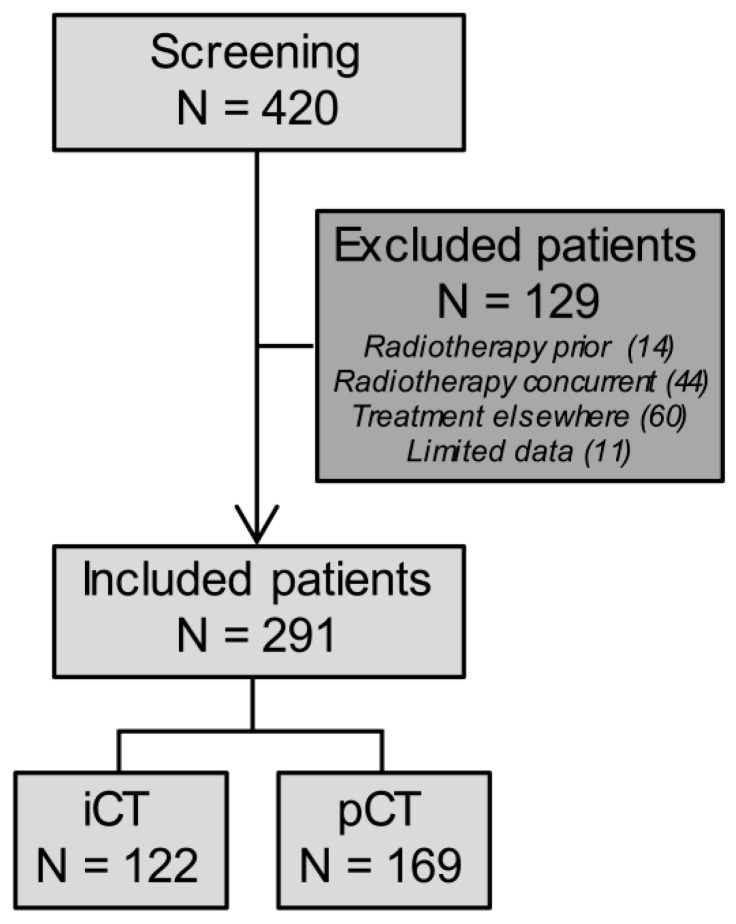
STROBE diagram of the included patients: Treatment elsewhere included patients who were referred for one or more cycles of chemotherapy in another hospital. Abbreviations: iCT = induction chemotherapy; N = number; pCT = palliative chemotherapy; STROBE = strengthening the reporting of observational studies in epidemiology.

A total of 420 patients with (gastro)esophageal cancer who were treated with carboplatin (AUC 4) and paclitaxel (100 mg/m^2^) were screened for inclusion of whom 129 patients were excluded, mainly because of concurrent radiotherapy or referral for chemotherapy in another hospital (Figure 1). This resulted in a total of 291 patients evaluable for the analysis of whom 122 patients were included in the induction chemotherapy (iCT) group and 169 patients in the palliative chemotherapy (pCT) group. For 8 patients (iCT: 3 and pCT: 5), the date of death was unknown, and they were excluded from the overall survival analysis.

The majority of patients in both groups were male (76% and 82%, respectively) and had a good WHO performance status (i.e., WHO 0 or 1). The median ages were 64 years (IQR: 58–69) in the iCT group and 61 years (IQR: 55–68) in the pCT group. Most patients still smoked tobacco and used alcohol before diagnosis or currently on a regular base. All patient and treatment characteristics are described in Table 1. In the iCT group, the incidence of the adenocarcinoma was almost equal to squamous cell carcinoma (50% versus 48%) in contrast to the pCT group where the incidence of adenocarcinoma was higher (70% versus 28%). In the iCT group, tumors were most often located in the distal esophagus (47%) and also present in the proximal (19%), middle esophagus (24%), and gastroesophageal junction (11%). In the pCT group, the majority of all tumors were located at the distal esophagus (73%). The UICC-AJCC TNM-stage was also different between the iCT and pCT patients, which resembles the intent of treatment per group, with more patients with metastatic disease and a higher variety of metastases locations in the pCT group (Table 1). The administered mean carboplatin and paclitaxel dose were comparable in both groups, and the median number of treatment cycles was 7 in the iCT group and 8 in the pCT group (i.e., six weekly cycles, followed by 3-weekly cycles).

### 2.2. Toxicity

Overall, this treatment regimen was relatively well-tolerated. However, the incidence of overall grade of ≥3 toxicity was 71% in the iCT group and 78% in the pCT group. These grade 3 or 4 toxicities occurred mainly as hematological toxicity, especially neutropenia with grade 3 in 43% and grade 4 in 25% of patients in both groups. However, febrile neutropenia occurred in ten patients only, resulting in a low incidence of 3% complicated neutropenia (Table 2). Gastrointestinal toxicity was mostly low-graded, and a gastrointestinal toxicity of grade ≤2 occurred mainly as nausea (iCT: 39%, pCT: 46%), constipation (iCT: 34%, pCT: 42%), and anorexia (iCT: 21%, pCT: 26%). Severe gastrointestinal toxicity (i.e., grade ≥3) occurred in 3% of the iCT and 5% of the pCT patients (Table 2). Fatigue of grade ≤2 occurred in 70% of patients of both groups, but severe fatigue occurred in only six patients. Neuropathy was mostly seen as low-graded sensory neuropathy in a quarter of patients in both groups and as motoric neuropathy grade 2 in six patients. Adverse events rarely resulted in a dose reduction of carboplatin or paclitaxel, as dose reductions were applied in only 2 patients due to severe nausea in one patient and febrile neutropenia in the other. Transfusion-related reactions were more frequent and occurred mainly as a reaction to paclitaxel (instead of carboplatin) in around one fifth of the patients. 

During the six weekly cycles, a cycle delay due to toxicity occurred in 42% and 43% of the patients treated with iCT and pCT, respectively. This delay was mainly caused by thrombocytopenia (46%), leukocytopenia (20%), or a combination of both (11%); other reasons included non-neutropenic fever (6%) and gastrointestinal toxicity (5%). A cycle delay consisted of one week in 70% of patients with a delay and two weeks in 19% of patients and occurred mostly at cycle 4 (70%). For patients with a cycle delay due to toxicity, this was often one cycle (85%); however, in 18 patients (15%), two separate cycles were delayed due to hematological toxicity. A premature termination of the planned six weekly cycles due to toxicity occurred in 14 iCT patients (12%) and 28 pCT patients (17%) mostly due to general malaise (43%), thrombocytopenia (14%) and leukocytopenia (10%), or both (7%). Hospitalization due to toxicity occurred in 18 iCT patients (15%) and 17 pCT patients (10%), which was caused by gastrointestinal toxicity in 11 patients, febrile neutropenia in 10 patients, non-neutropenic fever in 13 patients, and malaise in one patient. There were no toxic deaths in our cohort.

### 2.3. Efficacy

After six weekly cycles, the overall response rate was 48% for iCT and 44% pCT, with a complete response in 1% of both groups (Table 3). For many iCT patients, additional treatment was given: definitive chemoradiotherapy (7%), esophagectomy (35%), exploratory laparotomy (4%), or second line chemotherapy (17%) (Table 3). In the pCT group, some patients received another treatment as well: definitive chemoradiotherapy (1%) [29], esophagectomy (4%), exploratory laparotomy (5%), or second-line chemotherapy (19%).

The median follow-up for the 27 iCT patients who were still alive was 43.7 months (range of 2–117 months) and 18.4 months in 21 patients for pCT (range of 6–124 months). For the whole group of iCT patients, the median progession free survival (PFS) was 12.4 months (IQR: 7.1–45.3 months) and the median overall survival (OS) was 15.6 months (IQR: 9.7–36.3). However, in the 42% of patients who underwent a subsequent resection or radiotherapy median PFS was 22.1 months (IQR: 12.4–114.2) and the median OS was 26.8 months (IQR: 15.4–91.7). For patients treated with pCT, the median PFS was 8.2 months (IQR: 5.1–14.5) and the median OS 10.9 months (IQR: 6.5–18.3). The median PFS and OS for patients treated with pCT are comparable to iCT patients who did not receive an esophagectomy or definitive chemoradiotherapy afterwards (Table 3). 

### 2.4. Predictive and Prognostic Factors

In the iCT group, smoking was identified as a predictive factor for poor response (i.e., stable or progressive disease), with an odds ratio of 2.30 (95% confidence interval (CI): 1.02–2.21, P = 0.045) for current smokers compared to former and nonsmokers. Unfortunately, no other predictive factors for treatment response could be identified in the iCT group or in the pCT group; for these results, see Supplementary Table S1. 

For iCT patients, smoking was also an adverse prognostic factor for PFS and OS univariately but only remained significant in the multivariate analysis of PFS with an hazard ratio (HR) of 2.61 (95% CI: 1.17–5.85, P = 0.020) for current smokers versus nonsmokers. In addition, elevated thrombocyte number and alkaline phosphatase levels were also adverse prognostic factors for PFS in the multivariate model (HR: 1.00, 95% CI: 1.00–1.01, P = 0.001 and HR: 1.02, 95% CI: 1.00–1.03, P = 0.023, respectively). For OS, higher WHO performance status (HR: 1.87, 95% CI: 1.06–3.29, P = 0.031), T-stage (T4B; HR: 1.82, 95% CI: 1.02–3.25, P = 0.044), and thrombocyte number (HR: 1.00, 95% CI: 1.00–1.00, P = 0.025) were adverse prognostic factors in the multivariate model, see Table 4. 

For pCT patients, tumor location and year of diagnosis remained prognostic factors in the multivariate analysis for PFS with patients with a mid-esophageal tumor having a better PFS compared to proximal tumors (HR: 0.30, 95% CI: 0.11–0.83, P = 0.020). For OS, the mid-esophageal tumor location was also found to be significant with a comparable HR as for PFS. Other variables included in the multivariate model for OS were body surface area (BSA; HR: 0.34, 95% CI: 0.12–0.91, P = 0.032) and WHO 1/2 versus 0 (HR: 1.69, 95% CI: 1.13–2.52, P = 0.011). For all results, see Table 4.

## 3. Discussion

This retrospective analysis demonstrated that a weekly regimen of six cycles of carboplatin (AUC 4) and paclitaxel (100 mg/m^2^) is a relatively well-tolerated and an effective induction or palliative treatment for patients with advanced or metastatic (gastro)esophageal cancer. 

In general, the incidence of a toxicity grade ≥3 was low except for hematological toxicity. Almost two thirds of patients experienced severe neutropenia, which is higher than described for other frequently used regimens [22]. However, the incidence of severe leukocytopenia (i.e., leukocytes lower than 2.0 × 10^9^/L) was much lower than that of neutropenia observed and comparable with other frequently used regimens [22]. According to our treatment protocol, this regimen could be safely administered on an outpatient basis at any grade of neutropenia as long as the leukocyte number was sufficient (i.e., day 0 and 28: >3.0 × 10^9^/L; days 7, 14, 35, and 42: >1.0 × 10^9^/L), which was also reflected by the low incidence of febrile neutropenia we observed. 

Furthermore, the incidence of severe nausea or diarrhea of only one to two percent of patients was much lower than in most other regimens described using combinations with 5-fluorouracil or capecitabine [22]. In patients treated with fluorupyrimidine- and/or platinum-based triplets, severe nausea has been described in 7–21% and severe diarrhea has been described in 3–19% of patients [22,30]. Gastrointestinal toxicity is less when a doublet treatment is used compared to triplets but still seems higher than in our regimen, although no direct comparison could be made. In doublets where a fluoropyrimidine is combined with platinum, severe nausea occurred in 7–27% and severe diarrhea occurred in 4–8%, and with irinotecan, severe nausea occurred in 7% and severe diarrhea in 22%, respectively [22]. Polyneuropathy was mostly low graded and occurred in 4% of patients as grade 2 polyneuropathy, while grade 3 was not observed. For example, capecitabine combined with oxaliplatin resulted in 8% grade 3 polyneuropathy [31]. However, the incidence of severe hematological toxicity was only 4% with that regimen, while the incidence of complicated neutropenia was not mentioned [31]. Lastly, 22% of all patients developed an infusion-related reaction to paclitaxel, which is most likely caused by its formulation vehicle Cremophor [32]. However, this could usually easily be managed by prolonging the infusion time of paclitaxel. Infusion-related reactions to paclitaxel are not limited to this treatment regimen as much higher incidences up to 44% of patients treated with paclitaxel have been described [32]. The good clinical tolerance of this treatment regimen was also demonstrated by the low incidence of toxicity-related dose reductions and hospitalization. A premature end of treatment due to toxicity occurred more often and was mostly caused by general malaise, which is often multifactorial and therefore probably not (fully) caused by our regimen in all patients with a premature end of treatment. Furthermore, in palliative treatment, the quality of life should be considered as well, and a major advantage of the current regimen is that this treatment can be given as an outpatient treatment for which no hospitalization or central line is required, although this advantage is not limited to our treatment regimen. 

With this regimen, overall response rates of 48% in patients treated with induction intent and 44% in patients treated with palliative intent were achieved. However, as this analysis was not a randomized phase III study, all comparisons should be interpreted with caution as no direct comparison could be made. The percentages we found are slightly lower than the response rate of 54% found in our previous phase-1 study, which can be explained by the low sample size of only 37 patients in that former study and the difference in WHO performance of a selected study population compared to our cohort of non-selected patients [26]. The response rate of 48% in patients with an induction intent is comparable to other regimens with response rates varying between 20–48% (Table 5) [17,30,31,33,34,35,36,37,38,39,40,41]. However, most studies included a mixed population of patients with locally advanced and metastatic disease. Two studies did include only patients with locally irresectable disease without distant metastases and found a response rate of 32% for treatment with cisplatin and fluorouracil and of 45% for a treatment with docetaxel, cisplatin, and fluouracil [17,18]. However, these studies were conducted in patients with SCC, and our induction cohort consisted also for 50% of patients with an adenocarcinoma which makes it difficult to compare these results. Furthermore, for 42% of iCT patients, esophagectomy or definitive chemoradiotherapy followed, resulting in a median PFS of 22.1 months and a median OS of 26.8 months. For iCT patients, who did not have an esophagectomy or definitive chemoradiation, the median PFS of 9.0 months and OS of 11.8 months was lower but comparable to the patients treated with palliative intent. 

In metastatic (gastro)esophageal cancer, we found a response rate of 44% with our treatment regimen, which is also comparable with the most frequent used other doublet regimens (20–45%) and triplet regimens (31–48%) (Table 5) [30,31,33,34,35,36,37,39,40,41] When comparing survival rates, we found a median PFS of 8.2 months and OS of 10.9 months for palliative treated patients, which is longer than other doublet regimens in this patient group (PFS: 3.7–5.9 months; OS: 8.6–10.7) and is comparable to triplet regimens (PFS: 5.6–7.0; OS: 9.2–11.2) (Table 5) [30,31,33,34,35,36,37,39,40,41]. Although, we have to interpret these comparisons with caution as no direct comparison can be made, weekly carboplatin and paclitaxel seems at least equally effective and possibly even more effective compared to other frequently used treatment regimens and was better tolerated than other regimens. However, these results need further validation in a randomized phase-III trial with a direct comparison with other first-line treatment regimens.

As a secondary aim of the analysis, we tried to identify predictive and prognostic factors. We could only identify smoking as a predictive factor for patients with induction treatment, while unfortunately no predictive factors for treatment outcome in palliative treated patients were found. Nonetheless, we identified several prognostic factors for progression free survival and for overall survival. For induction chemotherapy, current smoking behavior, elevated thrombocyte number and alkaline phosphatase levels, WHO status, and T-stage were identified as adverse prognostic factors. For palliative chemotherapy, tumor location, BSA, and WHO status were identified as prognostic factors. We could not confirm other known prognostic factors for survival such as sex or location of metastases [8,42,43]. Interestingly, smoking behavior was identified as a negative predictive and prognostic factor. Several reasons for this can be hypothesized, including sarcopenia and factors related to this unhealthy lifestyle. However, the underlying mechanism is not yet unraveled.

Our study has some limitations which need to be mentioned. The retrospective nature of our study could have influenced the quality of the data and the selection of patients. Nevertheless, our patient population included all patients who were treated in a certain time period and hence can be considered as a real-world patient cohort and therefore also representative for daily clinical practice. The retrospective data collection will mainly influence the incidence of low-grade adverse events as they are not always recorded but has no effect on the higher-graded adverse events as they have more clinical consequences and were described in detail. Furthermore, we included patients who were considered unresectable by the multidisciplinary team based on general criteria, but we could not retrospectively retrieve if this decision was possibly (in part) based on certain comorbidities. Also, we could not retrieve data on the human epidermal growth factor receptor type 2 (HER2) expression of the tumor, as this was not determined in individual patients, which potentially could have influenced the results. Lastly, it was impossible to include a quality of life analysis, which is especially important in treatments with palliative intent.

In this retrospective cohort study, we demonstrated that weekly paclitaxel and carboplatin is an effective and well-tolerated treatment regimen, and after further validation, this could be a valid induction or palliative treatment option in advanced (gastro)esophageal cancer. Despite the fact that chemotherapy in general has a limited efficacy in esophageal cancer with only minor differences between different schedules, it will remain the backbone of treatment in metastatic (gastro)esophageal cancer until new treatments are developed. Future research should therefore focus on predictive factors and biomarkers to identify patients who will benefit from a certain treatment beforehand. Furthermore, the tumor biology should be included in patient selection. Several molecular subtypes of (gastro)esophageal cancer have been identified and provide a rationale to develop a tailored treatment for the different subtypes instead of treating all (gastro)esophageal cancers in the same manner [44]. Currently investigated targeted therapies focus on targeting the HER2 and the vascular endothelial growth factor receptor (VEGF) with limited effect; nevertheless, several combination therapies are being evaluated [45]. Lastly, the immune microenvironment of the tumor might be a possible treatment target, as demonstrated by the promising results of nivolumab and/or ipilimumab in recent phase-II studies [46,47]. Furthermore, the phase-III ATTRACTION trial demonstrated that nivolumab significantly increased OS compared to the placebo in heavily pretreated Asian patients with metastatic (gastro)esophageal cancer independent of the programmed death ligand-1(PD-L1) status [48]. The phase-III KEYNOTE-181 study demonstrated that pembrolizumab as a second-line therapy significantly improved OS compared to chemotherapy in patients with metastatic esophageal cancer with a high PD-L1 combined score [49]. Currently, the results of multiple clinical trials evaluating the combination of immunotherapy and chemotherapy are awaited [45].

In conclusion, we demonstrated in this retrospective analysis that weekly paclitaxel and carboplatin is an effective and well-tolerated induction or palliative treatment regimen in a real-life patient cohort. However, as this analysis was not a randomized phase-III trial, its true value for clinical practice need further validation. Therefore, future research should directly compare this treatment regimen with other first-line treatment options.

## 4. Materials and Methods 

The patient cohort for this analysis was obtained from the Erasmus University Medical Center, Rotterdam, The Netherlands. The primary end point was a treatment response in patients with (gastro)esophageal cancer treated with a weekly regimen of carboplatin (AUC 4) and paclitaxel (100 mg/m^2^) with an induction or palliative treatment intent. Secondary end points included PFS, OS, the identification of predictive or prognostic factors, and the evaluation of toxicity. As this treatment regimen was considered routine clinical care at the Erasmus University Medical Center, no specific ethics approval or informed consent was required to retrospectively collect and analyze these data for research purposes.

### 4.1. Patients

All patients with (gastro)esophageal cancer treated with a weekly regimen of carboplatin (AUC 4) and paclitaxel (100 mg/m^2^) between October 2002 and May 2018 at the Erasmus University Medical Center, Rotterdam, The Netherlands were identified by the hospital pharmacy based on drug-dispensing data and evaluated for inclusion. Patients were excluded if radiotherapy on the esophagus was given concurrent or prior to the start of treatment with weekly carboplatin and paclitaxel, if one or more cycles were given outside the Erasmus University Medical Center, or if there was limited data on investigated cycles recorded in the electronic patient file (e.g., due to missing paper files uploaded in the electronic patient file). A multidisciplinary team consisting of a medical oncologist, upper gastro-intestinal surgeon, gastroenterologist, radiologist, and a radiotherapist decided upon the treatment intent (induction or palliative treatment). Patients with unresectable disease due to advanced locoregional bulky disease and/or suspected lymph nodes outside the field of possible radiation therapy (i.e., around the common hepatic artery, splenic hilum, or caudal to the celiac artery) were considered for induction treatment. All patients with distant metastasis were considered for palliative treatment. The treatment intent as decided by the multidisciplinary team was used for all further analyses and not retrospectively altered.

### 4.2. Treatment

Treatment consisted of a weekly regimen of carboplatin (AUC 4) and paclitaxel (100 mg/m^2^) for three weeks, then one week of rest, followed by another three weekly cycles. After these six cycles, a response evaluation with a computed tomography (CT) scan followed. For patients with a regression of the primary tumor or a disappearance of distant metastases while not developing new distant metastases after these first six weekly cycles, another treatment option could follow (i.e., esophagectomy or definitive chemoradiotherapy [29]). The selection criteria for esophagectomy were as follows: a radical and curative resection was deemed possible, no distant metastases, sufficient clinical condition for surgery, and patient’s consent for surgery. Definitive chemoradiotherapy was proposed to all other patients with a good response after the first six weekly cycles. 

For patients without other treatment options (e.g., due to distant metastasis) with a good response on these first six weekly cycles, treatment was continued with three 3-week cycles of carboplatin (AUC 6) and paclitaxel (175 mg/m^2^). 

Paclitaxel and carboplatin were diluted in 500 mL of sodium chloride solution (0.9%), and both administered in a 1-h infusion. All patients received intravenous premedication consisting of dexamethasone 10 mg, ranitidine 50 mg, clemastine 2 mg within 30 min before paclitaxel infusion, and granisetron 1 mg administered within 30 min before carboplatin infusion [25]. Before the start of a new cycle, hematological laboratory values had to fulfill the following requirements: for the first and fourth cycle (day 0 and 28): leukocytes >3.0 × 10^9^/L and thrombocytes >100 × 10^9^/L and for the second, third, fifth, and sixth cycles (days 7, 14, 35, and 42): leukocytes >1.0 × 10^9^/L and thrombocytes >50 × 10^9^/L. There were no restrictions on the absolute neutrophil count. Furthermore, for a full paclitaxel dose, adequate transaminases and bilirubin were required (bilirubin ≤1.5× upper limit of normal (ULN) and aspartate-/ alanine aminotransferase (AST/ALT) ≤ 2.5 × ULN (in case of liver metastases: AST/ALT ≤ 5 × ULN)). 

### 4.3. Data

Data related to patient demographics, tumor characteristics, laboratory results, adverse events, treatment, response, and survival were collected. Tumors were (re)staged according to the 7th edition of the UICC-AJCC TNM staging manual [50]. Laboratory results and adverse events were scored according to the Common Terminology Criteria for Adverse Events (CTC-AE) version 4.03 [51]. All adverse events occurring during the six weekly cycles and up to one week after treatment were collected, and the highest grade per item was used in the analysis. Severe toxicity was defined as adverse events with a CTC-AE grade 3 or higher. Gastrointestinal toxicity was defined as the occurrence of anorexia, nausea, vomiting, diarrhea, constipation, and mucositis. Hematological toxicity was defined as the occurrence of anemia, thrombocytopenia, leukocytopenia, and neutropenia. 

Response was determined after six cycles on radiological evaluation by CT-scan according to the radiologist (and if possible) by the response evaluation criteria in solid tumours (RECIST) criteria [52]. If there was no radiological evaluation possible due to clinical deterioration or death, this was counted as progressive disease (PD) on date of whatever came first. PFS and OS were defined as the time from start of chemotherapy till date of radiological or clinical progression or death, respectively.

### 4.4. Statistical Analysis

For all analyses, the patient cohort was divided in patients treated with an induction or palliative treatment intent. These groups were analysed separately to prevent bias induced by baseline patient and tumor characteristics. Demographic characteristics and toxicity were described per group. Survival time (i.e., PFS and OS) was estimated according to the Kaplan–Meier method. Predictive factors for treatment response were analysed with a logistic regression analysis, where a good response was defined as a complete or partial response (CR or PR). A good response was compared to stable disease (SD) and progressive disease (PD) together. Prognostic factors for survival (PFS and OS) were identified by univariate and multivariate Cox regression analyses. All factors with a p-value < 0.1 detected in univariate analyses were included in multivariate analyses. A backward selection method was used for the multivariate model where a threshold of p < 0.05 was applied. In general, p-values < 0.05 were considered statistically significant. All statistical analyses were performed using Stata version 15.1 (StataCorp LP 2017. Statistical Software, College Station, Texas, USA). 

## 5. Conclusions

Our study demonstrates that six weekly cycles of carboplatin (AUC 4) and paclitaxel (100 mg/m^2^) is a well-tolerated and effective induction or palliative treatment regimen and, therefore, a good option for patients with locally advanced or metastatic disease. 

## Figures and Tables

**Table 1:** Patient, tumor, and treatment characteristics.

Characteristic	iCT (N = 122)	pCT (N = 169)
Sex		
Male	93 (76%)	138 (82%)
Female	29 (24%)	31 (18%)
Age (years)		
Median (IQR)	64 (58–69)	61 (55–68)
Performance Status		
WHO 0	28 (23%)	49 (29%)
WHO 1	73 (60%)	90 (53%)
WHO 2	7 (6%)	5 (3%)
Unknown	14 (12%)	25 (13%)
Ethnic Origin		
Caucasian	93 (76%)	120 (71%)
African	1 (0.8%)	3 (2%)
Asian	0	4 (2%)
Unknown	28 (23%)	42 (25%)
BSA (m^2^) ^a^		
Mean (SD)	1.91 (0.22)	1.89 (0.21)
eGFR (mL/min) ^b^		
Median (IQR)	93 (84–99)	92 (78–100)
Unknown	21 (17%)	45 (27%)
Smoking		
Never	15 (12%)	31 (18%)
Before diagnosis	22 (18%)	36 (21%)
Current	80 (66%)	91 (54%)
Unknown	5 (4%)	11 (7%)
Alcohol		
Never	24 (20%)	29 (17%)
Before diagnosis	54 (44%)	81 (48%)
Current	37 (30%)	48 (28%)
Unknown	7 (6%)	11 (7%)
Tumor Location		
Proximal	23 (19%)	8 (5%)
Middle	29 (24%)	26 (15%)
Distal	57 (47%)	123 (73%)
GE-junction	13 (11%)	10 (6%)
Multiple locations	0	2 (1%)
Tumor Type		
Adenocarcinoma	61 (50%)	117 (70%)
Squamous cell carcinoma	59 (48%)	48 (28%)
Other ^c^	2 (2%)	3 (2%)
Unknown	0	1 (1%)
Tumor Differentiation		
Good	7 (6%)	3 (2%)
Moderate	38 (31%)	50 (30%)
Poor	48 (39%)	74 (44%)
Unknown	29 (24%)	42 (25%)
T-stage		
T1b	0	3 (2%)
T2	5 (4%)	12 (7%)
T3	66 (54%)	112 (66%)
T4a	27 (22%)	23 (14%)
T4b	24 (20%)	13 (8%)
N-stage		
N0	12 (10%)	21 (12%)
N1	49 (40%)	69 (41%)
N2	48 (39%)	56 (33%)
N3	13 (11%)	23 (14%)
M-stage		
M0	71 (58%)	15 (9%)
M1	51 (42%)	155 (91%)
Metastases Location		
Lymph nodes	50 (41%)	72 (43%)
Liver	0	19 (11%)
Lungs	0	3 (2%)
Other	0	18 (11%)
Multiple locations	1 (1%)	42 (25%)
Not applicable	71 (58%)	15 (9%)
Disease Stage		
IB	1 (1%)	0
IIA	5 (4%)	2 (1%)
IIB	1 (1%)	2 (1%)
IIIA	17 (14%)	4 (2%)
IIIB	13 (11%)	2 (1%)
IIIC	34 (28%)	5 (3%)
IV	51 (42%)	154 (91%)
Carboplatin dose (mg)		
Mean (SD)	477 (90)	486 (95)
Paclitaxel dose (mg)		
Mean (SD)	191 (22)	187 (22)
Number of treatment cycles ^d^		
Median (IQR)	7 (6–9)	8 (6–9)

^a^ BSA was calculated according to the Mosteller formula [27]; ^b^ Estimated glomerular filtration rate (eGFR) was calculated according to the Chronic Kidney Disease Epidemiology Collaboration (CKD-EPI) formula [28]; ^c^ Tumor Type Other included undifferentiated large cell carcinomas and neuroendocrine carcinomas; ^d^ Six weekly cycles, followed by 3-weekly cycles. Abbreviations: BSA = body surface area; GE = gastro-esophageal; iCT = induction chemotherapy; IQR = inter-quartile range; mg = milligram; N = number; pCT = palliative chemotherapy; SD = standard deviation; WHO = World Health Organization.

**Table 2:** Toxicity and clinical consequences.

	iCT (N = 122)			pCT (N = 169)		
	Grade 1	Grade 2	Grade ≥3	Grade 1	Grade 2	Grade ≥3
**Overall Toxicity**	114 (93%)	111 (91%)	86 (71%)	167 (99%)	154 (91%)	131 (78%)
Gastrointestinal Toxicity						
Anorexia	21 (17%)	5 (4%)	1 (1%)	35 (21%)	9 (5%)	3 (2%)
Nausea	39 (32%)	8 (7%)	1 (1%)	68 (40%)	10 (6%)	3 (2%)
Vomiting	19 (16%)	4 (3%)	0	27 (16%)	5 (3%)	3 (2%)
Diarrhea	20 (16%)	1 (1%)	2 (2%)	21 (12%)	10 (6%)	2 (1%)
Constipation	31 (25%)	10 (8%)	0	57 (34%)	15 (9%)	0
Mucositis	4 (3%)	2 (2%)	0	17 (10%)	0	1 (1%)
Other Toxicity						
Alopecia	32 (26%)	40 (33%)	NA	50 (30%)	65 (39%)	NA
Dermatitis	9 (7%)	2 (2%)	0	10 (6%)	4 (2%)	0
Fatigue	59 (48%)	25 (21%)	1 (1%)	82 (49%)	36 (21%)	5 (3%)
Sensory Neuropathy	27 (22%)	3 (3%)	0	40 (24%)	4 (2%)	0
Motoric Neuropathy	1 (2%)	0	0	3 (2%)	6 (4%)	0
Hematological Toxicity						
Anemia	57 (47%)	58 (48%)	6 (5%)	86 (51%)	64 (38%)	17 (10%)
Thrombocytopenia	67 (55%)	22 (18%)	13 (11%)	83 (49%)	27 (16%)	22 (13%)
Leukocytopenia	7 (6%)	56 (46%)	43 (35%)	18 (11%)	74 (44%)	52 (31%)
Neutropenia	0	21 (17%)	82 (67%)	0	24 (14%)	113 (67%)
Other blood value alterations						
Creatinine Increase	6 (5%)	0	0	6 (4%)	0	0
AST Increase	21 (17%)	0	1 (1%)	29 (17%)	2 (1%)	4 (2%)
ALT Increase	20 (16%)	3 (3%)	0	20 (12%)	3 (2%)	2 (1%)
GGT Increase	20 (16%)	4 (3%)	1 (1%)	25 (15%)	14 (8%)	12 (7%)
AP Increase	19 (16%)	0	0	25 (15%)	1 (1%)	6 (4%)
Bilirubin Increase	12 (10%)	5 (4%)	0	7 (4%)	7 (4%)	3 (2%)
**Clinical Consequences**	**iCT (N = 122)**			**pCT (N = 169)**		
	**No**	**Yes: toxicity**	**Yes: other**	**No**	**Yes: toxicity**	**Yes: other**
Febrile Neutropenia	118 (97%)	4 (3%)	NA	163 (96%)	6 (4%)	NA
Dose reduction Carboplatin	117 (96%)	1 (1%)	3 (3%) ^a^	126 (75%)	1 (1%)	4 (2%) ^a^
Dose reduction Paclitaxel	97 (79%)	1 (1%)	24 (20%) ^a^	126 (75%)	1 (1%)	40 (24%) ^a^
Treatment delay	64 (53%)	51 (42%)	7 (6%)	84 (50%)	72 (43%)	11 (7%)
Premature end of treatment *	95 (78%)	14 (12%)	13 (11%) ^b^	109 (64%)	29 (17%)	31 (19%) ^b^
Hospitalization	96 (79%)	18 (15%)	8 (7%)	140 (83%)	17 (10%)	12 (7%)

* Premature end of treatment was defined as the end of treatment before the planned six weekly cycles. ^a^ Dose reduction other was defined as a transfusion-related reaction to paclitaxel or carboplatin; in these cases, the dose was not reduced but the infusion time was prolonged. ^b^ Premature end of treatment other included disease progression in 7 (iCT) and 16 (pCT) patients. Abbreviations: ALT = alanine aminotransferase; AP = alkaline phosphatase; AST = aspartate aminotransferase; GGT = gamma-glutamyltransferase; iCT = induction chemotherapy; N = number; NA = not applicable, pCT = palliative chemotherapy.

**Table 3:** Treatment response.

	iCT (N = 122)	pCT (N = 169)
Response after 6 cycles		
Complete Response	1 (1%)	2 (1%)
Partial Response	57 (47%)	72 (43%)
Stable Disease	46 (38%)	56 (33%)
Progressive Disease	11 (9%)	23 (14%)
Unknown	7 (6%)	16 (10%)
Treatment afterwards ^a^		
Carboplatin-Paclitaxel ^b^	13 (11%)	14 (8%)
Chemotherapy Other ^c^	8 (7%)	18 (11%)
Definitive Chemoradiotherapy ^d^	9 (7%)	1 (1%)
Esophagectomy	43 (35%)	7 (4%)
PFS (months; median (IQR))		
All patients	12.4 (7.1–45.3)	8.2 (5.1–14.5)
No CRT or esophagectomy afterwards	9.0 (4.3–13.4)	8.0 (5.0–13.2)
CRT or esophagectomy afterwards	22.1 (12.4–114.2)	18.1 (14.8–122.2)
OS (months; median [IQR])		
All patients	15.6 (9.7–36.3)	10.9 (6.5–18.3)
No CRT or esophagectomy afterwards	11.8 (7.3–18.6)	10.6 (6.4–17.2)
CRT or esophagectomy afterwards	26.8 (15.4–91.7)	23.1 (14.8–28.0)

^a^ Intervention after the last administration of carboplatin (AUC 4) and paclitaxel (100 mg/m^2^); ^b^ Second period of treatment with carboplatin (AUC 4) and paclitaxel (100 mg/m^2^); ^c^ Chemotherapy other than carboplatin and paclitaxel; including EOX (epirubicin, oxaliplatin, and capecitabine), 5-fluorouracil combined with cisplatin, or phase-1 trial medication combined with docetaxel, irinotecan, or capecitabine; ^d^ Definitive chemoradiotherapy included six weekly cycles of carboplatin (targeted at AUC 2) and paclitaxel (50 mg/m^2^) combined with radiotherapy on the esophagus [29]. Abbreviations: AUC = area under the curve; CRT = definitive chemoradiotherapy; iCT = induction chemotherapy; IQR = inter-quartile range; N = number; OS = overall survival; pCT = palliative chemotherapy; PFS = progression free survival.

**Table 4:** Prognostic factors for PFS and OS in patients treated with induction or palliative chemotherapy.

Baseline Factor	Induction Chemotherapy (iCT)												Palliative Chemotherapy (pCT)											
	Progression Free Survival						Overall Survival						Progression Free Survival						Overall Survival					
	Univariate Analysis			Multivariate Analysis			Univariate Analysis			Multivariate Analysis			Univariate Analysis			Multivariate Analysis			Univariate Analysis			Multivariate Analysis		
	HR	95% CI	*P*	HR	95% CI	*P*	HR	95% CI	*P*	HR	95% CI	*P*	HR	95% CI	*P*	HR	95% CI	*P*	HR	95% CI	*P*	HR	95% CI	*P*
**Sex (M vs. F)**	0.77	0.45–1.29	*0.319*				0.83	0.51–1.34	*0.443*				1.00	0.63–1.59	*0.989*				0.90	0.58–1.39	*0.623*			
**Age**	1.01	0.98–1.03	*0.600*				1.01	0.99–1.03	*0.496*				1.00	0.98–1.03	*0.738*				1.00	0.98–1.02	*0.892*			
**BSA**	0.89	0.30–2.62	*0.835*				0.63	0.24–1.64	*0.341*				0.80	0.33–1.94	*0.618*				0.30	0.13–0.69	*0.005*	0.34	0.12–0.91	*0.032*
**WHO (1 vs. 0)**	1.31	0.77–2.23	*0.322*				2.20	1.30–3.72	*0.003*	1.87	1.06–3.29	*0.031*	1.34	0.90–2.01	*0.154*				1.70	1.16–2.48	*0.006*	1.69	1.13–2.52	*0.011*
**Alcohol (vs never) History Current**	1.03 1.50	0.44–2.43 0.76–2.96	*0.311*				1.49 1.98	0.64–3.49 0.95–4.14	*0.114*				0.71 0.90	0.40–1.29 0.57–1.44	*0.498*				0.83 0.87	0.50–1.40 0.56–1.34	*0.767*			
**Smoking (vs never) History Current**	1.17 2.22	0.63–2.18 1.14–4.33	*0.032* *0.619* *0.019*	1.28 2.61	0.60–2.75 1.17–5.85	*0.522* *0.020*	1.40 2.42	0.76–2.60 1.26–4.63	*0.015* *0.281* *0.008*				0.92 1.02	0.55–1.53 0.58–1.78	*0.883*				0.92 0.98	0.57–1.47 0.59–1.63	*0.915*			
**Year of diagnosis**	1.03	0.96–1.10	*0.381*				1.01	0.95–1.08	*0.689*				1.06	1.01–1.12	*0.016*	1.06	1.01–1.12	*0.028*	1.01	0.96–1.05	*0.833*			
**Tumor location (vs proximal)** **Middle** **Junction/Cardia** **Multiple locations**	1.17 0.99 0.94 NA	0.57–2.43 0.52–1.91 0.37–2.41 NA	*0.937*				0.79 0.67 0.55	0.43–1.45 0.39–1.16 0.24–1.26	*0.429*				0.32 0.59 0.69 0.85	0.12–0.89 0.24–1.45 0.22–2.11 0.16–4.42	*0.100* *0.029* *0.246* *0.513* *0.849*	0.29 0.55 0.68 0.73	0.10–0.80 0.22–1.36 0.22–2.08 0.14–3.80	*0.017* *0.193* *0.498* *0.705*	0.27 0.45 0.46 0.87	0.12–0.63 0.22–0.94 0.18–1.17 0.18–4.13	*0.045* *0.002* *0.033* *0.103* *0.859*	0.27 0.54 0.49	0.11–0.65 0.26–1.15 0.18–1.33	*0.004* *0.110* *0.160*
**Histology (SCC vs. AC)**	1.06	0.66–1.68	*0.816*				1.22	0.80–1.86	*0.354*				0.56	0.36–0.86	*0.008*				0.76	0.52–1.10	*0.146*			
**Differentiation (poor vs. good/moderate)**	1.40	0.83–2.37	*0.206*				1.15	0.71–1.86	*0.572*				1.06	0.70–1.63	*0.776*				1.06	0.72–1.56	*0.761*			
**T-stage** **(iCT: vs. T2/T3, pCT: vsT1b/T2)** **T3** **T4A** **T4B**	NA 1.28 1.61	NA 0.74–2.22 0.86–3.00	*0.305*				NA 0.98 2.20	NA 0.57–1.68 1.32–3.66	*0.014* *NA* *0.948* *0.003*	NA 1.01 1.82	NA 0.56–1.81 1.02–3.25	NA *0.984* *0.044*	1.85 1.68 1.36	0.98–3.48 0.76–3.71 0.56–3.28	*0.204*				2.03 2.13 1.59	1.11–3.71 1.03–4.41 0.71–3.55	*0.076*			
**N-stage (vs N0)** **N1** **N2** **N3**	0.72 0.83 0.79	0.29–1.75 0.35–2.00 0.28–2.25	*0.888*				0.93 0.94 0.77	0.47–1.83 0.48–1.86 0.33–1.82	*0.935*				0.92 1.17 1.15	0.51–1.65 0.65–2.12 0.59–2.24	*0.704*				1.03 1.14 1.23	0.60–1.76 0.65–1.98 0.65–2.33	*0.867*			
**M-stage (vs M0)**	0.97	0.61–1.53	*0.880*				0.90	0.59–1.38	*0.634*				2.72	1.00–7.38	*0.050*				1.05	0.58–1.90	*0.877*			
**Metastases location:** **Nodal** **Liver** **Other** **Multiple locations**	NA	NA	NA				NA	NA	NA				1.78 3.43 2.36 2.51	0.71–4.48 1.26–9.33 0.85–6.57 0.98–6.44	*0.048* *0.219* *0.016* *0.099* *0.055*				1.00 1.26 1.14 1.16	0.54–1.86 0.57–2.77 0.54–2.42 0.60–2.23	*0.916*			
**Liver metastases (Y vs. No)**	NA	NA	NA				NA	NA	NA				1.47	1.02–2.13	*0.040*				1.12	0.78–1.60	*0.534*			
**Hemoglobin (mmoL/L)**	0.99	0.79–1.26	*0.961*				0.83	0.67–1.03	*0.087*				0.95	0.80–1.12	*0.520*				0.86	0.74–0.00	*0.052*			
**Thrombocytes (10^9^/L)**	1.00	1.00–1.00	*0.010*	1.00	1.00–1.01	*0.001*	1.00	1.00–1.00	*0.020*	1.00	1.00–1.00	*0.025*	1.00	1.00–1.00	*0.695*				1.00	1.00–1.00	*0.684*			
**Leukocytes (10^9^/L)**	1.05	0.97–1.13	*0.206*				1.04	0.97–1.11	*0.270*				1.01	0.99–1.03	*0.478*				1.00	0.99–1.02	*0.735*			
**Neutrophils (10^9^/L)**	1.05	0.96–1.14	*0.310*				1.04	0.96–1.12	*0.312*				1.01	0.97–1.05	*0.696*				1.01	0.95–1.08	*0.660*			
**ASAT (U/L)**	1.00	0.97–1.03	*0.958*				0.99	0.97–1.02	*0.599*				1.00	1.00–1.01	*0.145*				1.00	1.00–1.01	*0.883*			
**ALAT (U/L)**	1.00	0.99–1.01	*0.880*				1.00	0.99–1.01	*0.633*				1.00	1.00–1.00	*0.767*				1.00	0.99–1.00	*0.475*			
**LD (U/L)**	1.00	1.00–1.00	*0.396*				1.00	1.00–1.00	*0.716*				1.00	1.00–1.00	*0.908*				1.00	1.00–1.00	*0.414*			
**GGT (U/L)**	1.00	1.00–1.01	*0.301*				1.00	1.00–1.01	*0.193*				1.00	1.00–1.00	*0.608*				1.00	1.00–1.00	*0.973*			
**AP (U/L)**	1.01	1.00–1.01	*0.056*	1.02	1.00–1.03	*0.023*	1.00	1.00–1.01	*0.183*				1.00	1.00–1.00	*0.948*				1.00	1.00–1.00	*0.943*			
**Bilirubin (µmol/L)**	1.01	0.95–1.07	*0.836*				1.02	0.96–1.08	*0.596*				1.00	0.96–1.03	*0.953*				1.01	0.97–1.04	*0.722*			
**Kreatinin (µmol/L)**	0.99	0.97–1.00	*0.114*				1.00	0.98–1.01	*0.665*				1.00	0.99–1.02	*0.552*				0.99	0.98–1.01	*0.356*			

*P*-values < 0.05 are considered statistically significant and are depicted in bold. Abbreviations: AC = adenocarcinoma; ALT = alanine aminotransferase; AP = alkaline phosphatase; AST = aspartate aminotransferase; F = female; GGT = gamma-glutamyltransferase; HR = hazard ratio; iCT = induction chemotherapy; LD = lactate dehydrogenase; M = male; N = number; NA = not applicable OR = odds ratio; OS = overall survival; pCT = palliative chemotherapy; PFS = progression free survival; SCC = squamous cell carcinoma; vs = versus (reference category); World Health Organization Performance Status; Y = yes.

**Table 5:** Summary of the induction and palliative treatment regimens mentioned.

	Patients (N)	Age (Median)	Esophageal Tumor (%)	GEJ Tumor * (%)	Gastric Tumor (%)	Adenocarcinoma (%)	Locally Advanced (%)	Metastatic Disease (%)	Overall Response (%)	Median PFS (Months)	Median OS (Months)
Fluorouracil, Cisplatin [33]	224	55	0	25	75	90	3	97	25	3.7	8.6
Fluorouracil, Cisplatin [34]	163	59	0	19	81	100	5	95	26	4.2	8.7
Fluorouracil, Cisplatin [17]	163	56 ^a^	100	0	0	0	100	0	32	NR	11.0
Fluorouracil, Oxaliplatin [31]	51	60	NR	NR	0	88	NR	NR	39	5.3	8.0
Fluorouracil, Oxaliplatin [35]	64	63	100	0	0	0	0	100	41	4.0	10.0
Docetaxel, Cisplatin [41]	76	57	0	26	74	100	5	95	20	5.0	10.5
Paclitaxel, Cisplatin [36]	51	56	100	0	0	61	10	90	43	NR	9.0
Etoposide, Cisplatin [37]	73	60	100	0	0	0	4	96	45	NR	8.5
Epirubicin, Cisplatin, Fluorouracil [30]	263	65	35	29	36	90	21	80	41	6.2	9.9
Epirubicin, Cisplatin, Capecitabine [30]	250	64	30	28	42	90	23	77	46	6.7	9.9
Epirubicin, Oxaliplatin, Fluorouracil [30]	245	61	30	23	37	86	23	77	42	6.5	9.3
Epirubicin, Oxaliplatin, Capecitabine [30]	244	62	34	22	44	87	24	58	48	7.0	11.2
Docetaxel, Cisplatin, Fluorouracil [38]	48	66	100	0	0	0	81	19	31	17.6	NR ^b^
Docetaxel, Cisplatin, Fluorouracil [33]	221	55	0	19	81	89	3	96	37	5.6	9.2
Fluorouracil, Folinic Acid, Cisplatin [39]	108	64	0	22	78	100	9	91	25	3.9	8.8
Fluorouracil, Folinic Acid, Oxaliplatin [39]	112	64	0	18	82	100	3	97	35	5.8	10.7
Fluorouracil, Folinic Acid, Irinotecan [34]	170	58	0	20	80	100	4	96	32	5.0	9.0
Fluorouracil, Folinic Acid, Cisplatin, Etoposide [40]	69	55	100	0	0	0	19	81	34	NR	9.5

* If GEJ was not separately mentioned as tumor location, patients were grouped as esophageal tumor; ^a^ Mean age instead of median age; ^b^ Median overall survival was not reached; the 1-year survival rate was 67.9%; Abbreviations: GEJ = gastro-esophageal junction; N = number; NR = not reported; OS = overall survival; PFS = progression free survival.

## Supplementary Materials

The following are available online at https://www.mdpi.com/2072-6694/11/6/826/s1, Table S1: Predictive factors for treatment response in patients treated with induction or palliative chemotherapy.
