# Supplementary material for: Efficacy and Toxicity of Weekly Carboplatin and Paclitaxel as Induction or Palliative Treatment in Advanced Esophageal Cancer Patients
Source: Cancers (Basel). 2019 Jun 13;11(6):826. doi: 10.3390/cancers11060826 (PMC6627268; doi:10.3390/cancers11060826)
Supplement: Supplementary file 1 [file cancers-11-00826-s001.pdf]

## Supplementary Materialas

**Table S1.** Predictive factors for treatment response in patients treated with induction or palliative chemotherapy.

| Baseline Factors | Treatment Response (iCT) Univariate Analysis (CR/PR vs SD/PD) | | | Treatment Response (pCT) Univariate Analysis (CR/PR vs SD/PD) | | |
|---|---|---|---|---|---|---|
| | OR | 95% CI | *p*-value | OR | 95% CI | *p*-value |
| **Gender** (M vs F) | 0.89 | 0.37–2.10 | *0.786* | 0.84 | 0.37–1.89 | *0.672* |
| **AGE** | 1.01 | 0.97–1.05 | *0.666* | 0.98 | 0.94–1.01 | *0.207* |
| **BSA** | 0.99 | 0.19–5.08 | *0.993* | 0.65 | 0.15–2.87 | *0.566* |
| **WHO** (1 vs 0) | 1.19 | 0.50–2.83 | *0.702* | 0.86 | 0.42–1.76 | *0.678* |
| **Alcohol** (vs never/history | | | | | | |
| current | 1.48 | 0.66–3.29 | *0.341* | 0.93 | 0.47–1.80 | *0.820* |
| **Smoking** (vs never/history) | | | | | | |
| current | 2.30 | 1.02–2.21 | ***0.045*** | 0.84 | 0.41–1.73 | *0.634* |
| **Tumor location** (vs proximal) | | | | | | |
| Middle | 0.86 | 0.27–2.75 | *0.796* | 0.13 | 0.01–1.25 | *0.078* |
| Distal | 1.20 | 0.43–3.31 | *0.735* | 0.18 | 0.02–1.51 | *0.113* |
| Junction/Cardia | 0.50 | 0.42–2.40 | *0.361* | 0.10 | 0.01–1.29 | *0.077* |
| Multiple locations | | | | | | |
| **Histology** (SCC vs AC) | 0.72 | 0.35–1.52 | *0.394* | 1.00 | 0.49–2.01 | *0.990* |
| **Differentiation** | | | | | | |
| (poor vs good/moderate) | 1.19 | 0.52–2.76 | *0.680* | 0.62 | 0.29–1.32 | *0.215* |
| **T-stage** | | | | | | |
| (iCT: vs T2/T3, pCT: vs T1b/T2) | | | | | | |
| T3 | | | | 1.53 | 0.51–4.61 | *0.450* |
| T4A/B | 1.38 | 0.65–2.94 | *0.401* | 2.13 | 0.60–7.57 | *0.245* |
| **N-stage** (vs N0) | | | | | | |
| N1 | 1.71 | 0.43–6.91 | *0.449* | 0.80 | 0.30–2.16 | *0.657* |
| N2 | 1.57 | 0.40–6.27 | *0.527* | 0.78 | 0.28-2.19 | *0.639* |
| N3 | 0.94 | 0.17–5.07 | *0.940* | 0.68 | 0.19–2.35 | *0.537* |
| **M-stage** (vs M0) | 1.03 | 0.49–2.20 | *0.935* | 1.55 | 0.46–5.10 | *0.474* |
| **Metastases location** | | | | | | |
| (vs no metastases) | | | | | | |
| Nodal | | | | 2.11 | 0.62–7.16 | *0.229* |
| Liver | | | | 0.87 | 0.20–3.90 | *0.858* |
| Other | | | | 4.48 | 0.99–20.4 | *0.052* |
| Multiple locations | | | | 1.24 | 0.34–4.47 | *0.746* |
| **Liver metastases** (Y vs No) | | | | 0.67 | 0.34–1.33 | *0.254* |
| **Hemoglobin** (mmol/L) | 0.83 | 0.57–1.20 | *0.313* | 1.01 | 0.76–1.35 | *0.921* |
| **Thrombocytes** ($10^9$/L) | 1.00 | 1.00–1.00 | *0.487* | 1.00 | 0.99–1.00 | *0.121* |
| **Leukocytes** ($10^9$/L) | 1.05 | 0.92–1.19 | *0.462* | 0.99 | 0.95–1.03 | *0.560* |
| **Neutrophils** ($10^9$/L) | 1.04 | 0.89–1.20 | *0.641* | 0.97 | 0.91–1.05 | *0.487* |
| **ASAT** (U/L) | 0.98 | 0.94–1.03 | *0.449* | 1.00 | 0.99–1.01 | *0.590* |
| **ALAT** (U/L) | 1.00 | 0.98–1.02 | *0.824* | 1.00 | 0.99–1.01 | *0.455* |
| **LD** (U/L) | 1.00 | 1.00–1.00 | *0.794* | 1.00 | 1.00–1.00 | *0.807* |
| **GGT** (U/L) | 1.00 | 0.98–1.01 | *0.535* | 1.00 | 1.00–1.00 | *0.631* |
| **AP** (U/L) | 1.01 | 0.99–1.03 | *0.220* | 1.00 | 1.00–1.00 | *0.413* |
| **Bilirubin** (μmol/L) | 0.99 | 0.90–1.10 | *0.910* | 1.00 | 0.93–1.07 | *0.967* |
| **Kreatinin** (μmol/L) | 1.01 | 0.99–1.04 | *0.163* | 1.01 | 0.99–1.03 | *0.276* |

P-values < 0.05 are considered statistically significant and are depicted in bold. Abbreviations: AC = adenocarcinoma; ALAT = alanine aminotransferase; AP = alkaline phosphatase; ASAT = aspartate aminotransferase; F = female; GGT = gamma-glutamyltransferase; iCT = induction chemotherapy; LD = lactate dehydrogenase; M = male; N = number; OR = odds ratio; pCT = palliative chemotherapy; SCC =

squamous cell carcinoma; vs = versus (reference category); WHO = World Health Organization Performance Status; Y = yes.
